# Model error propagation in a compatible tree volume, biomass, and carbon prediction system

**DOI:** 10.1186/s13021-025-00303-6

**Published:** 2025-06-10

**Authors:** James A. Westfall, Philip J. Radtke, David M. Walker, John W. Coulston

**Affiliations:** 1 U.S. Forest Service, Northern Research Station, York, PA USA; 2 Virginia Tech, College of Natural Resources and Environment, Blacksburg, VA USA; 3 U.S. Forest Service, Southern Research Station, Blacksburg, VA USA

**Keywords:** Tree taper, Specific gravity, Carbon fraction, Volume ratio, Residual variance

## Abstract

**Background:**

Individual tree attributes such as volume, biomass and carbon mass are widely known to be highly correlated. As these attributes are typically predicted from statistical models, frameworks that provide compatible relationships among these attributes are usually preferred over approaches that provide independent predictions. However, the propagation of model error can be a concern as this compatibility often relies on predictions for one attribute providing the basis for other attributes. In this study, a compatible tree volume, biomass, and carbon prediction system was evaluated to ascertain how model prediction uncertainty propagates through the system and to examine the contribution to uncertainty in population estimates.

**Results:**

Generally, the total and merchantable stem volume predictions are used to derive associated biomass values and subsequently biomass is converted to carbon. As expected, the amount of uncertainty due to the models follows volume < biomass < carbon such that the carbon attribute is the most affected by error propagation. Biomass and associated carbon in tree branches tended to have larger model uncertainty than the stem components due to smaller sample sizes and a greater proportion of unexplained variation. In this model system, direct predictions of whole tree biomass provide the biomass basis and stem and branch components are harmonized to sum to the whole tree value. Corresponding harmonized carbon content values are obtained through application of a common carbon fraction. As such, whole tree biomass and carbon tended to have less model uncertainty than the constituent components primarily due to fewer contributing sources.

**Conclusions:**

Although a wide range of outcomes are realized across the various volume, biomass, and carbon components, increases in the standard error of the population estimate due to model uncertainty were always less than 5% and usually smaller than 3%. Thus, forest inventory data users desiring population estimates of tree volume, biomass, and carbon can expect little additional uncertainty due to the prediction model system while benefitting from the implicit compatibility among attributes.

## Background

Individual tree volume, biomass, and carbon attributes often provide the basis for estimating important forest resources. Estimates of tree volume are used for a myriad of purposes, including wood supply analyses [12, 17], forest management planning [3, 16], and wood product determination [4, 24]. Knowledge of tree biomass resources provide critical information for bioenergy [14] and biodiversity [1] assessments. Similarly, estimating amounts of carbon stored in forest trees is critical to understanding the role forests play in offsetting greenhouse gas emissions [15, 34]. It is considered a desirable property that these attributes are harmonious to avoid inconsistencies such as a large sound volume value being associated with a relatively small amount of biomass. As such, methods that produce compatible predictions of individual tree volume, biomass, and carbon attribute are often employed [37, 38].

Typically, tree volume, biomass, and carbon values are predicted from statistical models where sources of model error take the form of residual variance and parameter estimate uncertainty [20]. In addition to these models, wood specific gravity [23] and carbon fractions [8] may also be used to obtain desired predictions. In practice, these factors are often treated as known values but are actually mean values from samples of trees and therefore also subject to uncertainty. Point estimation and associated uncertainty of attributes where observations are predictions from statistical models have become increasingly recognized as ‘hybrid inference’ [6, 7, 10, 13, 27, 35]. In addition to these studies, a number of authors have examined the contributions of model error in the context of overall uncertainty pertaining to population estimates of tree volume, biomass, and/or carbon resources without specific reference to hybrid methods [5, 11, 19, 21, 28, 30].

One aspect that deserves further attention is understanding model propagation error when several prediction system component models are used. McRoberts et al. [22] examined the additional uncertainty in tree carbon estimates due to a volume prediction model that was subsequently scaled to carbon content via estimated wood specific gravity and carbon fraction means. The recently implemented approach for the national forest inventory (NFI) of the U.S. also uses various models/means to obtain both whole tree and subcomponent volume, biomass, and carbon predictions [36]. Thus, the contribution of different error sources may depend on the specific attribute being estimated. For example, whole stem volume is determined from a single allometric model, but prediction of merchantable stem volume requires additional predictions from both a taper model and a volume ratio model. Thus, it may be expected that merchantable stem volume is subject to more uncertainty than whole stem volume. Similarly, bark biomass is predicted directly from a regression model whereas stem wood biomass is obtained from conversion of predicted volume via wood specific gravity. Further uncertainty is introduced due to a total tree biomass prediction model and the harmonization of biomass component additivity to achieve compatibility. The objectives of this study are to 1) present and quantify the various sources of uncertainty and their roles within the prediction framework, 2) assess the effects of these uncertainty sources on overall precision of population estimates generated from the NFI data.

## Methods

### Forest inventory data

The data used to assess model error propagation were collected by the Forest Inventory and Analysis (FIA) program as part of the NFI of the U.S. Due to the lengthy computer processing time needed to implement the analytical methods described below, the domain was limited to white pine (*Pinus strobus*) trees in the state of Vermont. The data were collected over the period 2013–2019 and the sample consists of 1124 plots having the response design described by Bechtold and Scott [2]. A total of 864 live trees having diameter at breast height (dbh) ≥ 2.5 cm (1.0 in.) were used for assessments of total stem volume inside-bark (TSV^IB^), total stem bark volume (TSV^BK^), total stem volume outside-bark (TSV^OB^), total stem biomass inside-bark (TSB^IB^), total stem bark biomass (TSB^BK^), total stem biomass outside-bark (TSB^OB^), total stem carbon inside-bark (TSC^IB^), total stem bark carbon (TSC^BK^), total stem carbon outside-bark (TSC^OB^), branch biomass (B^BR^), branch carbon (C^BR^), total aboveground biomass (B^AG^), and total aboveground carbon (C^AG^). A subset of 848 trees with dbh ≥ 12.7 cm (5 in.) were used for analyses of merchantable stem (0.30 m (1 ft) stump height to 10.16 cm (4 in.) top diameter outside bark) volumes (MSV^IB^, MSV^BK^, MSV^OB^), biomass (MSB^IB^, MSB^BK^, MSB^OB^), and carbon (MSC^IB^, MSC^BK^, MSC^OB^). Volume, biomass, and carbon attributes were predicted for each tree using the methods described in Westfall et al. [36].

### Model fitting data

For the volume and biomass models, the raw data was necessary to model the residual variance as well as account for variances/covariances associated with the estimated coefficients. The white pine data obtained from Radtke et al. [31] included tree diameter at breast height (D), total height (H), and various volume (TSV^IB^, TSV^BK^, TSV^OB^), volume ratio (proportion of the total stem volume contained in the portion of the stem below height h ≤ H; R^IB^, R^OB^), biomass (B^BK^, B^BR^, B^AG^), and wood specific gravity (WDSG) information. Analysis of the carbon fraction (CF) for white pine used data from the carbon database described in Doraisame et al. [8]. The mean value for WDSG was $\overline{WDSG }$ = 0.338, which compares closely with the value of 0.34 used by the FIA program [23]. The standard error was ${\sigma }_{\overline{WDSG} }$ = 0.004. Due to the same data source being used, the CF mean of $\overline{CF }$ = 0.507 (${\sigma }_{\overline{CF} }$ = 0.003) is identical to that implemented by FIA [36]. To aid in reference for subsequent sections, Table 1 provides a listing of abbreviations and definitions. Table 2 provides a summary of the observed data.

**Table 1:** Summary of relevant abbreviations used throughout the manuscript

TSV^IB^ (MSV^IB^)	Total (Merchantable) stem volume inside-bark	CF	Carbon fraction
TSV^BK^ (MSV^BK^)	Total (Merchantable) stem bark volume	R^IB^	Volume ratio inside-bark
TSV^OB^ (MSV^OB^)	Total (Merchantable) stem volume outside-bark	R^OB^	Volume ratio outside-bark
TSB^IB^ (MSB^IB^)	Total (Merchantable) stem biomass inside-bark	TSBH^IB^ (MSBH^IB^)	Harmonized total (merchantable) stem biomass inside-bark
TSB^BK^ (MSB^BK^)	Total (Merchantable) stem bark biomass	TSBH^BK^ (MSBH^BK^)	Harmonized total (merchantable) stem bark biomass
TSB^OB^ (MSB^OB^)	Total (Merchantable) stem biomass outside-bark	TSBH^OB^ (MSBH^OB^)	Harmonized total (merchantable) stem biomass outside-bark
TSC^IB^ (MSC^IB^)	Total (Merchantable) stem carbon inside-bark	TSCH^IB^ (MSCH^IB^)	Harmonized total (merchantable) stem carbon inside-bark
TSC^BK^ (MSC^BK^)	Total (Merchantable) stem bark carbon	TSCH^BK^ (MSCH^BK^)	Harmonized total (merchantable) stem bark carbon
TSC^OB^ (MSC^OB^)	Total (Merchantable) stem carbon outside-bark	TSCH^OB^ (MSCH^OB^)	Harmonized total (merchantable) stem carbon outside-bark
B^BR^	Branch biomass (outside-bark)	BH^BR^	Harmonized branch biomass (outside-bark)
C^BR^	Branch carbon (outside-bark)	CH^BR^	Harmonized branch carbon (outside-bark)
B^AG^	Aboveground biomass (outside-bark)	WDSGH	Harmonized wood specific gravity
C^AG^	Aboveground carbon (outside-bark)	BKSGH	Harmonized bark specific gravity
WDSG	Wood specific gravity

**Table 2:** Sample size and distribution statistics for white pine model fitting data

Attribute	n	Min	Mean	Max	Std. dev.
D (cm)	3639	2.540	31.790	111.760	15.773
H (m)	3639	2.682	20.197	48.158	7.181
TSV^IB^ (m^3^)	2783	0.001	0.907	8.074	0.996
TSV^BK^ (m^3^)	2597	0.000	0.146	1.746	0.160
TSV^OB^ (m^3^)	3409	0.001	1.210	18.186	1.474
B^BK^ (kg)	392	0.113	42.598	310.121	54.444
B^BR^ (kg)	338	0.163	87.805	1010.199	146.403
B^AG^ (kg)	239	0.859	426.379	3695.632	584.860
R^IB^	39509^a^	0.004	0.626	1.000	0.337
R^OB^	54757^a^	0.004	0.617	1.000	0.339
WDSG	96	0.197	0.338	0.431	0.004^b^
CF	6	0.497	0.507	0.519	0.003^b^

^a^Number of stem profile observations

^b^Standard deviation of the mean

### Analysis

The model forms for white pine specified in Westfall et al. [36] were used for the analysis. Specifically, these formulations for component predictions were (1) for TSV^OB^, B^BR^, and B^AG^, (2) for TSV^BK^ and B^BK^, (3) for TSV^IB^, and (4) for R^IB^ and R^OB^:

1 $${y}_{i}=a*{D}_{i}^{b}*{H}_{i}^{c}+{\varepsilon }_{i}$$

2 $${y}_{i}=\left\{\begin{array}{c}a * {D}_{i}^{b} * {H}_{i}^{c}+{\varepsilon }_{i};{D}_{i}< k\\ a * {k}^{\left(b-{b}_{1}\right)} *{D}_{i}^{{b}_{1}} * {H}_{i}^{c}+{\varepsilon }_{i};{D}_{i}\ge k\end{array}\right.$$

3 $${y}_{i}=a*{D}_{i}^{b}*{H}_{i}^{c}*{exp}^{(-{(b}_{1}*{D}_{i}))}+{\varepsilon }_{i}$$

4 $${y}_{i}={\left(1-{\left(1-\frac{{h}_{i}}{{H}_{i}}\right)}^{\alpha }\right)}^{\beta }+{\varepsilon }_{i}$$

For tree *i*, *D*_*i*_ = diameter breast height (cm), *H*_*i*_ = total tree height (m), *h*_*i*_ = height along the stem (m), *ɛ*_*i*_ = residual error, *y*_*i*_ = observation for component of interest, *k* = segmentation point that is 22.86 cm (9 in.) for white pine, and *a*, *b*, *c*, *b*_*1*_, *α*, *β* are model-specific parameters estimated using the observed data described in Table 2. Model (4) is used to partition whole-stem predictions into merchantable-stem predictions using a lower bound based on a predefined stump height of 0.30 m (1 ft) and an upper bound corresponding with height *h*_*i*_ to the 10.16 cm (4 in.) top diameter outside-bark. The upper bound is obtained by recognizing that Model (4) can be combined with model (1) to estimate the height *h*_*i*_ to any diameter *d*_*i*_. The stem volume from groundline to *h*_*i*_ is the product of a total outside bark volume model and a volume ratio model:

5 $${y}_{i}=a*{D}_{i}^{b}*{H}_{i}^{c}* {\left(1-{\left(1-\frac{{h}_{i}}{{H}_{i}}\right)}^{\alpha }\right)}^{\beta }+{\varepsilon }_{i}$$

The implied taper function is then specified as [39]:

6 $${d}_{i}^{2}=a\times {D}_{i}^{b}*{H}_{i}^{c}/0.000078540/{H}_{i}\times \alpha \times \beta \times {\left(1-\frac{{h}_{i}}{{H}_{i}}\right)}^{\left(\alpha -1\right)}\times {\left(1-{\left(1-\frac{{h}_{i}}{{H}_{i}}\right)}^{\alpha }\right)}^{\left(\beta -1\right)}+{\varepsilon }_{i}$$

Thus, the desired height along the stem (*h*_*i*_) at a specified merchantable top diameter of *d*_*i*_ = 10.16 cm can be predicted by iteratively solving (7) for *h*_*i*_ via numeric optimization [25]:

7 $${0 \approx d}_{i}-(a\times {D}_{i}^{b}*{H}_{i}^{c}/0.000078540/{H}_{i}\times \alpha \times \beta \times {\left(1-\frac{{h}_{i}}{{H}_{i}}\right)}^{\left(\alpha -1\right)}\times {\left(1-{\left(1-\frac{{h}_{i}}{{H}_{i}}\right)}^{\alpha }\right)}^{\left(\beta -1\right)}{)}^{0.5}$$

Note that the total stem outside bark volume model is only used to provide parameter estimates *a*, *b*, and *c* for [7]. It is not used to directly predict outside bark volume as doing so would not preserve additivity properties between stem wood and bark components.

**Fig. 1:**
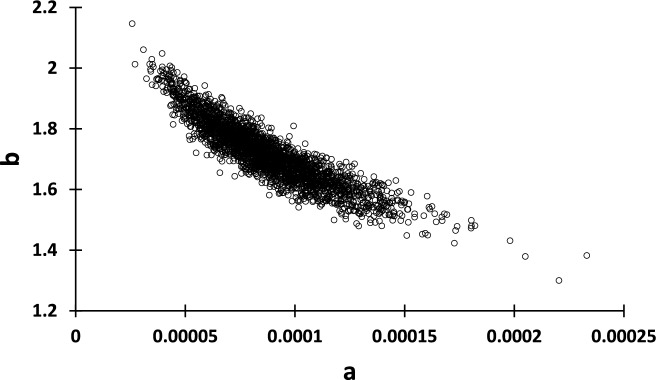
Scatterplot of model parameters *a* and *b* for TSV^IB^ model (3) from 2500 bootstrap samples

Quantification of model error using the destructively sampled tree data (Table 2) generally followed the methods described in McRoberts et al. [22]. In preparation for model error propagation simulations, correlated distributions of model parameters and models to predict residual variance were needed. For each of the volume and biomass models, the variances and covariances of parameter estimates were approximated by (1) drawing a bootstrap sample [9], (2) fitting the model to the bootstrap sample, and (3) recording the resulting parameter estimates. This process was repeated 2500 times for each model to provide a comprehensive range of possible parameter estimates (Fig. 1) for use in error propagation simulation analyses. Residual error behavior for models (1)–(4) were obtained by (1) ordering from smallest to largest the observations in the data, (2) creating *q* = *1* to* Q* equal-sized sequential groups having at least 25 observations each, (3) using the mean value from the bootstrap exercise for each model parameter, calculating the mean of the predicted values ${\widehat{\overline{y}} }_{q}$ and the standard deviation of the residuals ${\widehat{\sigma }}_{r{es}_{q}}$ in each group *q*, and (4) modeling the residual variance using (8) for models (1)–(3) and (9) for model (4) where ${\varnothing }_{p}$ (*p* ≤ 3) are estimated parameters and ${\varepsilon }_{q}$ is random residual error for group *q*.

8 $${\widehat{\sigma }}_{r{es}_{q}}= {\varnothing }_{1}{{\widehat{\overline{y}} }_{q}}^{{\varnothing }_{2}}+{\varepsilon }_{q}$$

9 $${\widehat{\sigma }}_{r{es}_{q}}= {\varnothing }_{1}{{\varnothing }_{2}}^{{({\widehat{\overline{y}} }_{q}-{\varnothing }_{3})}^{2}}+{\varepsilon }_{q}$$

The regression outcomes for predicting ${\widehat{\sigma }}_{r{es}_{q}}$ are provided in Table 3. Uncertainty in WDSG was assessed via the standard error of the mean (${\sigma }_{\overline{WDSG} }=0.004$). Similarly, the standard error of the mean (${\sigma }_{\overline{CF} }=0.003$) was used to represent CF variability (Table 2).

**Table 3:** Parameter estimates with standard errors in parenthesis and fit statistics for models (8) and (9)

Attribute	Model	∅_1_	∅_2_	∅_3_	R^2^	RMSE
TSV^IB^	(8)	0.1075 (0.0031)	0.9667 (0.0248)		0.987	0.018
TSV^BK^	(8)	0.2898 (0.0209)	1.0904 (0.0622)		0.968	0.010
TSV^OB^	(8)	0.1127 (0.0054)	0.7578 (0.0286)		0.955	0.039
R^IB^	(9)	0.0470 (0.0014)	0.0012 (0.0005)	0.5603 (0.0081)	0.961	0.006
R^OB^	(9)	0.0434 (0.0015)	0.0018 (0.0008)	0.5508 (0.0097)	0.962	0.005
B^BK^	(8)	0.4017 (0.0755)	0.8195 (0.0393)		0.997	0.781
B^BR^	(8)	1.8372 (0.2755)	0.6593 (0.0257)		0.997	2.819
B^AG^	(8)	0.1189 (0.0163)	0.9827 (0.0190)		0.999	1.575

### Uncertainty simulation

Assessments of additional uncertainty in population estimates due to model error were performed using the NFI data from Vermont in Monte Carlo simulations. The steps outlined below were taken for each replication *r* where *r* = 1 to 1500 was sufficient to obtain stable outcomes (maximum variation for *r* ≥ 1400 was about 0.5%). For each tree *i* in the forest inventory dataset, total inside bark stem wood volume ${\widehat{TSV}}_{i(r)}^{IB}$ was predicted using model (3) using a randomly drawn set of model parameters generated from the bootstrap samples described above. Residual uncertainty was incorporated by modifying ${\widehat{TSV}}_{i(r)}^{IB}$ as ${\widehat{TSV}}_{i(r)}^{IB\in }={\widehat{TSV}}_{i(r)}^{IB}+{z}_{(r)}\times {\widehat{\sigma }}_{res}$ where ${\widehat{TSV}}_{i(r)}^{IB\in }$ is the prediction that includes model uncertainty, ${z}_{(r)}$ is a random value from a N(0,1) distribution constrained within |${z}_{(r)}$|≤ 3.0 and ${\widehat{\sigma }}_{res}$ is obtained from model (8) substituting ${\widehat{TSV}}_{i(r)}^{IB}$ for $\widehat{\overline{y} }$. An analogous process was used for predictions of total bark volume ${\widehat{TSV}}_{j(r)}^{BK}$, where model (2) was used in conjunction with a randomly selected parameter set and application of residual variance per (8) to ultimately arrive at a predicted value of ${\widehat{TSV}}_{i(r)}^{BK\in }$. Total stem outside bark volume that includes model uncertainty was then obtained as ${\widehat{TSV}}_{i(r)}^{OB\in }={\widehat{TSV}}_{i(r)}^{IB\in }+ {\widehat{TSV}}_{i(r)}^{BK\in }$.

Associated estimates of biomass for these components are also subject to model error. At each iteration *r*, the uncertainty in WDSG is provided by ${WDSG}_{(r)}^{\in } =\overline{WDSG }+{z}_{(r)}\times {\sigma }_{\overline{WDSG} }$ where *z*_(*r*)_ is generated as described above. In the case of ${\widehat{TSV}}_{i(r)}^{IB\in }$, the WDSG uncertainty is accounted for in the conversion to mass (kg) via ${\widehat{TSB}}_{i(r)}^{IB\in }= {\widehat{TSV}}_{i(r)}^{IB\in }\times {WDSG}_{(r)}^{\in } \times 1000 kg/{m}^{3}$. The biomass of the bark ${\widehat{TSB}}_{i(r)}^{BK}$ is generated using model form (2), with parameter estimate and residual uncertainty being incorporated through the methods shown earlier to obtain the predicted ${\widehat{TSB}}_{i(r)}^{BK\in }$. Total stem outside bark biomass was then obtained as ${\widehat{TSB}}_{i(r)}^{OB\in }={\widehat{TSB}}_{i(r)}^{IB\in }+ {\widehat{TSB}}_{i(r)}^{BK\in }$. Finally, the uncertainty in the CF is incorporated as ${CF}_{(r)}^{\in } =\overline{CF }+{z}_{(r)}\times {\sigma }_{CF}$ (analogously to the ${WDSG}_{(r)}^{\in }$ method) and carbon predictions for total stem inside-bark ${\widehat{TSC}}_{i(r)}^{IB}$ and bark ${\widehat{TSC}}_{i(r)}^{BK}$ that include model uncertainty are respectively ${\widehat{TSC}}_{i(r)}^{IB\in }={\widehat{TSB}}_{i(r)}^{IB\in }\times {CF}_{(r)}^{\in }$ and ${\widehat{TSC}}_{i(r)}^{BK\in }={\widehat{TSB}}_{i(r)}^{BK\in }\times {CF}_{(r)}^{\in }$. Subsequently, total stem outside-bark carbon content is ${\widehat{TSC}}_{i(r)}^{OB\in }={\widehat{TSC}}_{i(r)}^{IB\in }+{\widehat{TSC}}_{i(r)}^{BK\in }$.

In addition to the various attributes and components associated with the tree stem, predictions for branch biomass and carbon may also be of interest as well as providing the supplementary information needed for total aboveground biomass and carbon predictions (excluding foliage) as a sum of the components. The methods described above for introducing parameter estimate and residual variance uncertainty were also applied to branch biomass predictions from model (1) to obtain ${\widehat{B}}_{i(r)}^{BR\in }$. For purposes explained below, model predictions are directly obtained for total aboveground biomass ${\widehat{B}}_{i(r)}^{AG\in }$ from (1) with parameter estimate and residual variance uncertainty incorporated in the usual fashion. Corresponding branch and aboveground carbon content predictions are respectively ${\widehat{C}}_{i(r)}^{BR\in }={\widehat{B}}_{i(r)}^{BR\in }\times {CF}_{(r)}^{\in }$ and ${\widehat{C}}_{i(r)}^{AG\in }={\widehat{B}}_{i(r)}^{AG\in }\times {CF}_{(r)}^{\in }$.

Due to strength of the model fit for predicting total aboveground biomass B^AG^, a constraint was imposed on the biomass (and subsequent carbon) predictions outlined above. The original prediction with model uncertainty for each biomass component (i.e., ${\widehat{TSB}}_{i(r)}^{IB\in }$, ${\widehat{TSB}}_{i(r)}^{BK\in }$, and ${\widehat{B}}_{i(r)}^{BR\in }$) was proportionally scaled to harmonize the component sum with the predicted aboveground total to enforce ${\widehat{TSBH}}_{i(r)}^{IB\in }+{\widehat{TSBH}}_{i(r)}^{BK\in }+{\widehat{BH}}_{i(r)}^{BR\in }={\widehat{B}}_{i(r)}^{AG\in }$ where ${\widehat{TSBH}}_{i(r)}^{IB\in }$, ${\widehat{TSBH}}_{i(r)}^{BK\in }$, and ${\widehat{BH}}_{i(r)}^{BR\in }$ are the harmonized components. Predictions of harmonized component carbon are then based on the corresponding harmonized biomass values, e.g., ${{\widehat{TSCH}}_{i(r)}^{IB\in }=\widehat{TSBH}}_{i(r)}^{IB\in }\times {CF}_{(r)}^{\in }$. In all applications of introducing random error to predictions, the magnitude of the errors was sufficiently small such that no negative values were produced.

To maintain compatibility of biomass and carbon predictions for portions of the main stem, calculations of ‘realized’ wood and bark specific gravity are needed:

$${{\widehat{WDSGH}}_{i(r)}^{\in }=\widehat{TSBH}}_{i(r)}^{IB\in }/{\widehat{TSV}}_{i(r)}^{IB\in }/1000$$

$${\widehat{BKSGH}}_{i(r)}^{\in }={\widehat{TSBH}}_{i(r)}^{BK\in }/{\widehat{TSV}}_{i(r)}^{BK\in }/1000$$

These values are of particular importance for obtaining harmonized predictions of components such as merchantable stem wood ${\widehat{MSBH}}_{i(r)}^{IB\in }$ and bark biomass ${\widehat{MSBH}}_{i(r)}^{BK\in }$. A harmonized outside-bark biomass ${\widehat{MSBH}}_{i(r)}^{OB\in }$ can also be obtained if desired.

$${\widehat{MSBH}}_{i(r)}^{IB\in }={\widehat{MSV}}_{i(r)}^{IB\in }\times {\widehat{WDSGH}}_{i(r)}^{\in }\times 1000$$

$${\widehat{MSBH}}_{i(r)}^{BK\in }={\widehat{MSV}}_{i(r)}^{BK\in }\times {\widehat{BKSGH}}_{i(r)}^{\in }\times 1000$$

$${\widehat{MSBH}}_{i(r)}^{OB\in }={\widehat{MSBH}}_{i(r)}^{IB\in }+{\widehat{MSBH}}_{i(r)}^{BK\in }$$

Although the primary use of adjusted specific gravity for this study is the merchantable stem component, harmonized biomass values for any portion of the stem can be obtained in combination with (4) and (7). Corresponding carbon content predictions of ${\widehat{MSCH}}_{i(r)}^{IB\in }$, ${\widehat{MSCH}}_{i(r)}^{BK\in }$, and ${\widehat{MSCH}}_{i(r)}^{OB\in }$ were calculated by multiplying the harmonized biomass by the carbon fraction ${CF}_{(r)}^{\in }$.

Population estimates of totals for the components described above were generated at each iteration *r*. The methods are described in Scott et al. (2005) where post-stratification is employed to reduce sampling variance. Observations for each sample plot *j* in post-stratum *h* (generically expressed as *y*_*hj(r)*_) are calculated by expanding the tree component value of interest to a per hectare basis and summing those values across all trees on the plot. Simple random sample estimators are used in each post-stratum *h* to obtain the component mean ${\overline{y} }_{h(r)}$ and variance of the mean $v\left({\overline{y} }_{h(r)}\right)$.

10 $${\overline{y} }_{h(r)}=\frac{{\sum }_{j}^{{n}_{h}}{y}_{hj(r)}}{{n}_{h}}$$

11 $$v\left({\overline{y} }_{h(r)}\right)=\frac{{\sum }_{j}^{{n}_{h}}{\left({y}_{hj(r)}-{\overline{y} }_{h(r)}\right)}^{2}}{{n}_{h}\left({n}_{h}-1\right)}$$

Provided *y*_*hj*_ is the observation for plot *j* in post-stratum *h* and *n*_*h*_ is the number of plots in post-stratum *h*. Estimates of the population total *y* and corresponding variance *v(y)* are calculated by combining across post-strata:

12 $${y}_{(r)}={A}_{T}\sum_{h=1}^{H}{W}_{h}{\overline{y} }_{h(r)}$$

13 $$v\left({y}_{(r)}\right)=\frac{{A}_{T}^{2}}{n}\left[\sum_{h=1}^{H}{W}_{h}{n}_{h}v\left({\overline{y} }_{h(r)}\right)+\sum_{h=1}^{H}(1-{W}_{h})\frac{{n}_{h}}{n}v\left({\overline{y} }_{h(r)}\right)\right]$$

Assigning *A*_*T*_ as the total population area, *W*_*h*_ as the post-stratum weights, and *n* as the total sample size. Over the *r* simulation iterations, the reported value of the population estimate (*y*) and the variance (*v*_*y*_) due to sampling are respectively determined from:

14 $$y=\frac{\sum_{r=1}^{1500}{y}_{(r)}}{1500}$$

15 $${v}_{y}=\frac{\sum_{r=1}^{1500}{v(y}_{(r)})}{1500}$$

The standard error of the estimated *y* is ${se}_{y}=\sqrt{{v}_{y}}$. The additional uncertainty due to the models *v*_*m*_ is the variance among the *y*_*(r)*_ calculated from:

16 $${v}_{m}=\frac{\sum_{r=1}^{1500}{{(y}_{(r)}-y)}^{2}}{1500-1}$$

Subsequently, the total variance is ${v}_{t}={v}_{y}+{v}_{m}$ with an associated standard error ${se}_{t}=\sqrt{{v}_{t}}$. The percentage increase in the standard error due to *v*_*m*_ is:

17 $${m}_{\%}=\frac{{se}_{t}-{se}_{y}}{{se}_{y}}100$$

Users of forest inventory data might also judge estimate reliability in the form of a sampling error that is typically expressed as the standard error relative to the estimate as a percentage, i.e., $\frac{{se}_{y}}{y}100$. Also reported is an indication of change in the sampling error statistic due to model error (${s}_{\%}$):

18 $${s}_{\%}=\frac{{se}_{t}-{se}_{y}}{y}100$$

## Results

The results of the regression analyses for attributes usings models (1)–(4) revealed good fits of the models to the observed data with (pseudo-) R^2^ ≥ 0.93 in all cases (Table 4). The poorest results were achieved for prediction of TSV^BK^ and B^BR^ where R^2^ < 0.95 was attained. Thus, in practice it may be expected that these models would contribute relatively larger amounts of uncertainty to population estimates than the other models with better performance.

**Table 4:** R^2^ statistic and summary of residual distribution for regression models used in the compatible prediction system

Response variable	Model	R^2^	Residual distribution			
			Min	Mean	Max	Std. dev.
TSV^IB^ (m^3^)	(3)	0.989	− 1.22	0.00	1.26	0.14
TSV^BK^ (m^3^)	(2)	0.949	− 0.36	0.00	0.80	0.08
TSV^OB^ (m^3^)	(1)	0.992	− 1.50	0.00	1.56	0.17
B^BK^ (kg)	(2)	0.973	− 80.59	0.07	55.06	11.26
B^BR^ (kg)	(1)	0.939	− 160.47	3.49	297.55	41.88
B^AG^ (kg)	(1)	0.989	− 345.90	8.30	359.66	73.95
R^IB^	(4)	0.998	− 0.163	0.002	0.203	0.030
R^OB^	(4)	0.998	− 0.147	0.002	0.213	0.028

For the inside- and outside-bark stem volume components (MSV^IB^, MSV^OB^, TSV^IB^, TSV^OB^), uncertainty due to the regression model resulted in ${m}_{\%}$ of about 0.2–0.4% (Table 5). However, larger effects were seen for the bark volumes (MSV^BK^, TSV^BK^) where ${m}_{\%}$ was greater than 0.7%. This outcome is likely due to the poorer bark volume model fit as compared to the inside-bark volume model (Table 3). Two insights are taken from these results: 1) the total model error *v*_*m*_ associated with MSV^IB^ and MSV^BK^ is slightly larger than the respective amounts for TSV^IB^ and TSV^BK^ due to the need for two additional volume ratio model (4) predictions to obtain MSV^IB^ and MSV^BK^, and 2) although the bark volume model (2) uncertainty is relatively large compared to that from the inside-bark volume model (3), there is little effect on standard errors for estimates of MSV^OB^ (= MSV^IB^ + MSV^BK^) and TSV^OB^ (= TSV^IB^ + TSV^BK^).

**Table 5:** Population estimates (*y*) (14), sampling variances (*v*_*y*_) (15), variances due to model uncertainty (*v*_*m*_) (16), percent increases in standard error due to model uncertainty (${m}_{\%}$) (17), and percent increases in sampling error due to model uncertainty (${s}_{\%}$) (18)

Component	*y*	*v*_*y*_	*v*_*m*_	*m*_*%*_	*s*_*%*_
MSV^IB^	3.34E + 07	1.31E + 13	9.48E + 10	0.36%	0.04%
MSBH^IB^	1.83E + 08	3.92E + 14	1.05E + 13	1.33%	0.14%
MSCH^IB^	9.29E + 07	1.01E + 14	2.99E + 12	1.47%	0.16%
MSV^BK^	5.25E + 06	3.26E + 11	5.61E + 09	0.86%	0.09%
MSBH^BK^	2.82E + 07	9.18E + 12	5.19E + 11	2.79%	0.30%
MSCH^BK^	1.43E + 07	2.36E + 12	1.42E + 11	2.96%	0.32%
MSV^OB^	3.87E + 07	1.75E + 13	1.13E + 11	0.32%	0.03%
MSBH^OB^	2.11E + 08	5.21E + 14	1.33E + 13	1.27%	0.14%
MSCH^OB^	1.07E + 08	1.34E + 14	3.82E + 12	1.42%	0.15%
TSV^IB^	3.52E + 07	1.43E + 13	6.60E + 10	0.23%	0.02%
TSBH^IB^	1.20E + 10	1.67E + 18	4.52E + 16	1.34%	0.14%
TSCH^IB^	6.10E + 09	4.30E + 17	1.29E + 16	1.49%	0.16%
TSV^BK^	5.54E + 06	3.59E + 11	5.46E + 09	0.76%	0.08%
TSBH^BK^	1.86E + 09	3.93E + 16	2.21E + 15	2.77%	0.30%
TSCH^BK^	9.42E + 08	1.01E + 16	5.97E + 14	2.91%	0.31%
TSV^OB^	4.08E + 07	1.92E + 13	7.21E + 10	0.19%	0.02%
TSBH^OB^	1.37E + 10	2.17E + 18	2.77E + 16	0.64%	0.07%
TSCH^OB^	6.96E + 09	5.58E + 17	8.89E + 15	0.79%	0.09%
BH^BR^	3.52E + 09	1.68E + 17	1.42E + 16	4.14%	0.48%
CH^BR^	1.79E + 09	4.33E + 16	3.76E + 15	4.25%	0.49%
B^AG^	1.74E + 10	3.50E + 18	6.45E + 16	0.92%	0.10%
C^AG^	8.83E + 09	9.01E + 17	1.91E + 16	1.06%	0.11%

The contribution of *v*_*m*_ to *v*_*t*_ for estimates of harmonized component biomass arise in various ways. In common to all components is the variability induced by the total above ground biomass model parameter and residual uncertainty that affects the total biomass prediction used as the basis to harmonize the constituent biomass components. This uncertainty also influences the calculations of adjusted wood and bark specific gravity values that are used to obtain harmonized merchantable stem biomass predictions of ${\widehat{MSBH}}_{i(r)}^{IB}$, ${\widehat{MSBH}}_{i(r)}^{BK}$, and ${\widehat{MSBH}}_{i(r)}^{OB}$. Similarly, additional uncertainty for the harmonized total stem inside-bark biomass component (TSBH^IB^) is due to conversion of volume to mass via WDSG. Table 5 suggests that uncertainty in WDSG produces about 4–5 times greater *m*_*%*_ and *s*_*%*_ than attributed to the inside-bark volume predictions. The total stem bark component (TSBH^BK^) is also subject to uncertainty arising from the model used to directly predict bark biomass. Uncertainty due to the bark biomass predictions resulted in *m*_*%*_ and *s*_*%*_ exceeding 3 times larger than those for prediction of bark volume. The outside-bark biomass component TSBH^OB^ has combined uncertainty due to TSV^IB^ conversion to mass via WDSG and bark biomass model prediction, however these components have smaller *m*_*%*_ and *s*_*%*_ increases than their constituent inside-bark and bark components due to *v*_*y*_ being a larger proportion of *v*_*t*_ for MSB^OB^ and TSB^OB^ (Table 5).

There was a relatively large increase in the standard error (*m*_*%*_ = 4.14) and sampling error (*s*_*%*_ = 0.48) for estimates of B^BR^ due to the prediction model uncertainty. Table 4 shows the fit statistics for the bark biomass model were mediocre relative to the other models being used and thus larger amounts of uncertainty are expected. It is shown that *m*_*%*_ = 0.92% and *s*_*%*_ = 0.10% for B^AG^, which is intermediate with *m%* = 0.64 (*s*_*%*_ = 0.07%) associated with the compositional component TSB^OB^ and the *m*_*%*_ = 4.14 (*s*_*%*_ = 0.48%) attributed to B^BR^ component as a consequence of *v*_*y*_ being a larger proportion of *v*_*t*_ for B^AG^.

Conversions of biomass to carbon content via CF showed similar outcomes to those described above such as components derived as sums of other components having smaller *m*_*%*_ and *s*_*%*_. For most of the carbon components *m*_*%*_ and *s*_*%*_ were about 1.1–1.2 times larger than for associated biomass estimates (Table 5). However, due to the relatively large *v*_*y*_ for branch estimates, the CF uncertainty produced *m*_*%*_ and *s*_*%*_ for C^BR^ that were only 1.03 times larger than for B^BR^. Over all components and attributes, with the exception of estimates for B^BR^ and C^BR^, *m*_*%*_ was less than 3% and *s*_*%*_ was often less than 0.30%. Whereas *m*_*%*_ and *s*_*%*_ were respectively about 1.0% and 0.10% for the key attributes of B^AG^ and C^AG^.

## Discussion

Numerous models were employed to provide a suite of predictions for tree volume, biomass, and carbon attributes. Due to the myriad data needed to accommodate the spectrum of desired outputs, information from various sources were combined to maximize sample sizes for each component. As such, the array of component predictions for biomass (and subsequently carbon) that arise from these independent data sources petitions for harmonization of component summations with direct predictions of total aboveground amounts. In this case it was chosen to proportionally scale the individual component predictions, but other approaches could have been taken to enforce harmonization such as partitioning the total into the components [29], constraining one component via subtraction [37], or using a different modeling strategy, e.g., seemingly unrelated regression (SUR [26]). For each approach there are advantages and disadvantages from both practical and statistical perspectives. Generally, the SUR approach has been shown to be the most efficient in terms of minimizing the standard errors of the parameter estimates [32]. It may be inferred that using SUR likely provides smaller *m*_*%*_ and *s*_*%*_ than reported here using the proportional harmonization method (assuming unbalanced data among components could be appropriately accounted for). As such, practitioners may consider evaluating various potential approaches to developing the overall prediction system to determine which method best suits their desired objectives.

Further clarification is warranted regarding the smaller *m*_*%*_ and *s*_*%*_ outcomes for components obtained as sums of subcomponents whose *m*_*%*_ and *s*_*%*_ were greater, e.g., TSV^OB^. As TSV^OB^ = TSV^IB^ + TSV^BK^, an alternative method of estimating TSV^OB^ would be to separately estimate population totals for TSV^IB^ and TSV^BK^ and sum those results. An examination of Table 4 shows equivalent estimates for the population total (*y*) would be obtained, but equivalence in estimated sampling variance (*v*_*y*_) would not be obtained by summing the component variances because the component estimates are not independent. Given the *v*_*y*_ for TSV^IB^, TSV^BK^, and TSV^OB^ are 5.07E + 14, 1.27E + 13, and 6.78E + 14 respectively (Table 5), it is clear that a positively valued covariance between TSV^IB^ and TSV^BK^ is present at the sample plot level. Also, the relationship between the estimated value and the sampling error is essentially the same for all three attributes. Thus, while the *v*_*m*_ are largely additive, the contribution of *v*_*y*_ becomes a larger proportion of *v*_*t*_ in the case of TSV^OB^ (and other outside bark components) and thus the effect of model uncertainty as expressed by *m*_*%*_ and *s*_*%*_ is smaller.

At the individual-tree level, relatively small amounts of total stem volume, biomass, and carbon are attributable to the bark component and similarly for branches as a component of whole tree biomass and carbon. For example, on average a 10% increase in the bark or branch component increases the proportion of whole stem bark by about 1.3% and similarly a 2.1% increase in branch proportion of aboveground biomass or carbon. As such, uncertainty in predictions for bark and branch components have relatively little effect on the whole stem or whole tree uncertainty. Despite the small contribution of bark and branches to whole stem/tree amounts, some users may be interested in obtaining estimates specifically for these components. For the bark component, volume and biomass are each directly predicted from a regression model. There was less uncertainty associated with bark volume predictions than bark biomass, largely due to the much smaller sample size and larger residual variance for bark biomass (Table 4). As such, data users can expect model uncertainty effects to be smaller for bark volume (*m*_*%*_ ≈ 0.8, *s*_*%*_ ≈ 0.1) than for bark biomass (*m*_*%*_ ≈ 2.8, *s*_*%*_ ≈ 0.3). Conversion of bark biomass to carbon content produce *m*_*%*_ and *s*_*%*_ about 1.1 times larger than those for biomass. The branch biomass model also had a relatively small sample size and large residual variance such that users considering model uncertainty effects on population estimate reliability can expect *m*_*%*_ ≈ 4.1 and *s*_*%*_ ≈ 0.48. Subsequent conversion to branch carbon incurs *m*_*%*_ ≈ 4.3 and *s*_*%*_ ≈ 0.49. Thus, consumers of branch biomass and carbon estimates may consider model uncertainty to be nontrivial and modify accordingly their assessment of population estimate reliability based on sampling uncertainty alone.

As implied by the notation, typical applications of $\overline{WDSG }$ and $\overline{CF }$ are as mean values derived from a sample of trees. Thus, variation in these attributes is appropriately expressed as standard errors of the mean. In that light, perhaps the weakest model in this study was for $\overline{CF }$, where only 6 observations were used to estimate the mean and standard error. It appears that these data may underestimate the uncertainty (${\sigma }_{\overline{CF} }$ = 0.003) as Lamlom and Savidge [18] reported a standard deviation for white pine CF of 0.16 but the sample size was not explicitly reported. If the data used in this study had a comparable standard deviation, it would imply a ${\sigma }_{\overline{CF} }$ of approximately 0.016 assuming n = 96 (Table 2). Conversely, the ${\sigma }_{\overline{WDSG} }$ = 0.004 corresponds well with results for softwood species ranging from 0.004–0.007 based on 60 observations [33]. The results indicate a notable increase in *m%* and *s*_*%*_ attributable to the WDSG role in conversion of volume to biomass, yet very little additional uncertainty accrues from conversion of biomass to carbon content (Table 5). Thus, the uncertainty contribution from CF may be underestimated, but this speculation requires further investigation that can only be conducted when additional CF information becomes available.

In this study, the model residual uncertainties were treated independently even though in some cases correlated errors were present. The complexity in accounting for these correlations arises from the data structure, which is comprised of observations from various independent studies with the information available being dependent on the specific study goals. Thus, there is not a consistent set of data used across different attribute prediction models. This situation is exemplified in Table 2 where it is shown the sample sizes differ among the models for TSV^IB^, TSV^BK^, and TSV^OB^, i.e., some trees in the data had only TSV^IB^ information, others may have had only TSV^OB^ data, and some had both TSV^IB^ and TSV^OB^ information. It is also clear from Table 2 that only a small proportion of data are in common between the TSV attributes (*n* > 2500) and B attributes (*n* < 400). In other cases, the data may be independent among models and no correlated errors exist, e.g., WDSG and CF. Nonetheless, readers should be aware of this technical aspect and further work is needed to incorporate these correlations into the analysis and examine the effects on model error propagation.

## Conclusion

Assessing error propagation when using a complex system of models is necessary to understand potentially nontrivial contributions to total uncertainty. For practical purposes, this study was performed using a narrow species and spatial domain. However, the methods illustrate the model error propagation pathways within the compatible volume, biomass, and carbon system while the results indicate the magnitude of additional uncertainty to be expected for the analysis undertaken. Generally, users of FIA volume, biomass, and carbon data can be assured the model uncertainty contributes little to reducing the reliability of the estimates beyond sampling variability alone. There may be situations where the model uncertainties are larger than reported here, such as in situations where models underlying a specific estimate are based on small samples and/or have relatively poor fit to the data.

Although substantial increases in efforts to collect data that support tree biomass and carbon prediction have occurred, the desire for extensive data that adequately covers the many species likely to be encountered in a large area forest inventory remains largely unrealized. Thus, there is opportunity for regression model improvements for various species and/or tree components such as bark and branches. Given their role as direct multipliers, WDSG and CF should not be overlooked as fields for potentially considerable gains in both accurate quantification and reduced uncertainty. In particular, researchers are encouraged to publish raw data for these attributes as only mean values for many species are currently provided in the literature.

## Data Availability

The data used for developing the prediction models were provided by Radtke et al. (2023) and are available at https://doi.org/10.7294/22582432. The forest inventory data is available from the FIA program at https://research.fs.usda.gov/products/dataandtools/tools/fia-datamart.

## References

[1] Ali A, Yan ER. The forest strata-dependent relationship between biodiversity and aboveground biomass within a subtropical forest. For Ecol Manage. 2017;401:125–34.

[2] Bechtold WA, Scott CT. The forest inventory and analysis plot design. Gen. Tech. Rep. SRS-80. Asheville, NC: U.S. Department of Agriculture, Forest Service, Southern Research Station; 2005, p. 37–52.

[3] Bettinger P, Boston K, Siry JP, Grebner DL. Forest management and planning. 2nd ed. London, UK: Academic Press; 2017.

[4] Brandeis TJ, Hartsell AJ, Bentley JW, Brandeis C. Economic dynamics of forests and forest industries in the Southern United States. Gen. Tech. Rep. SRS-152. Asheville, NC: US Department of Agriculture Forest Service, Southern Research Station; 2012.

[5] Breidenbach J, Antón-Fernández C, Petersson H, McRoberts RE, Astrup R. Quantifying the model-related variability of biomass stock and change estimates in the Norwegian National Forest Inventory. For Sci. 2014;60:25–33.

[6] Bullock EL, Healey SP, Yang Z, Acosta R, Villalba H, Insfrán KP, Melo JB, Wilson S, Duncanson L, Næsset E, Armston J. Estimating aboveground biomass density using hybrid statistical inference with GEDI lidar data and Paraguay’s national forest inventory. Env Res Lett. 2023;18: 085001.

[7] Corona P, Fattorini L, Franceschi S, Scrinzi G, Torresan C. Estimation of standing wood volume in forest compartments by exploiting airborne laser scanning information: model-based, design-based, and hybrid perspectives. Can J For Res. 2014;44:1303–11.

[8] Doraisami M, Kish R, Paroshy NJ, Domke GM, Thomas SC, Martin AR. A global database of woody tissue carbon concentrations. Sci Data. 2022;9:284.

[9] Efron B, Tibshirani RJ. An introduction to the bootstrap. New York: Chapman and Hall/CRC; 1994.

[10] Fortin M, Manso R, Schneider R. Parametric bootstrap estimators for hybrid inference in forest inventories. Forestry. 2018;91:354–65.

[11] Fu Y, Lei Y, Zeng W, Hao R, Zhang G, Zhong Q, Xu M. Uncertainty assessment in aboveground biomass estimation at the regional scale using a new method considering both sampling error and model error. Can J For Res. 2017;47:1095–103.

[12] Gc S, Potter-Witter K, Pokharel R, Leefers L, Norris P, Huff ES. Assessing the wood-basket and characterizing Michigan’s logging businesses by their reliance on nonindustrial private forests for stumpage. For Pol Econ. 2023;156: 103067.

[13] Gozé L, Ekström M, Sandring S, Jonsson BG, Wallerman J, Ståhl G. Estimation of plant density based on presence/absence data using hybrid inference. Ecol Inform. 2024;80: 102377.

[14] He L, English BC, Daniel G, Hodges DG. Woody biomass potential for energy feedstock in United States. J For Econ. 2014;20:174–91.

[15] Hoover CM, Smith JE. Current aboveground live tree carbon stocks and annual net change in forests of conterminous United States. Carb Bal Manage. 2021;16:17. 10.1186/s13021-021-00179-2 PMC8138985 34018077

[16] Jokela EJ, Martin TA, Vogel JG. Twenty-five years of intensive forest management with southern pines: important lessons learned. J For. 2010;108:338–47.

[17] Korhonen KT, Ahola A, Heikkinen J, Henttonen HM, Hotanen JP, Ihalainen A, Melin M, Pitkänen J, Räty M, Sirviö M, Strandström M. Forests of Finland 2014–2018 and their development 1921–2018. Silva Fennica. 2021;55:5.

[18] Lamlom SH, Savidge RA. A reassessment of carbon content in wood: variation within and between 41 North American species. Biomass Bioenergy. 2003;25:381–8.

[19] Lin J, Gamarra JG, Drake JE, Cuchietti A, Yanai RD. Scaling up uncertainties in allometric models: how to see the forest, not the trees. For Ecol Manage. 2023;537: 120943.

[20] McRoberts RE, Westfall JA. Effects of uncertainty in model predictions of individual tree volume on large area volume estimates. For Sci. 2014;60:34–42.

[21] McRoberts RE, Westfall JA. Propagating uncertainty through individual tree volume model predictions to large-area volume estimates. Ann For Sci. 2016;73:625–33.

[22] McRoberts RE, Chen Q, Domke GM, Ståhl G, Saarela S, Westfall JA. Hybrid estimators for mean aboveground carbon per unit area. For Ecol Manage. 2016;378:44–56.

[23] Miles PD, Smith WB. Specific gravity and other properties of wood and bark for 156 tree species found in North America. Res. Note NRS-38. Newtown Square, PA: U.S. Department of Agriculture, Forest Service, Northern Research Station; 2009.

[24] Murphy G, Lyons J, O’Shea M, Mullooly G, Keane E, Devlin G. Management tools for optimal allocation of wood fibre to conventional log and bio-energy markets in Ireland: a case study. Eur J For Res. 2010;129:1057–67.

[25] Nocedal J, Wright SJ. Numerical optimization. 2nd ed. New York, NY: Springer Science + Business Media; 2006.

[26] Parresol BR. Additivity of nonlinear biomass equations. Can J For Res. 2001;31(5):865–78.

[27] Patterson PL, Healey SP, Ståhl G, Saarela S, Holm S, Andersen HE, Dubayah RO, Duncanson L, Hancock S, Armston J, Kellner JR. Statistical properties of hybrid estimators proposed for GEDI—NASA’s global ecosystem dynamics investigation. Env Res Lett. 2019;14: 065007.

[28] Petersson H, Breidenbach J, Ellison D, Holm S, Muszta A, Lundblad M, Ståhl GR. Assessing uncertainty: sample size trade-offs in the development and application of carbon stock models. For Sci. 2017;63:402–12.

[29] Poudel KP, Temesgen H. Methods for estimating aboveground biomass and its components for Douglas-fir and lodgepole pine trees. Can J For Res. 2016;46(1):77–87.

[30] Qin L, Meng S, Zhou G, Liu Q, Xu Z. Uncertainties in above ground tree biomass estimation. J For Res. 2021;32:1989–2000.

[31] Radtke P, Walker D, Frank J, Weiskittel A, MacFarlane D, Affleck D, Temesgen H, Poudel K, Zhao D, Auty D, Sánchez Meador A, Shaw J, Gray A, Westfall J, Coulston J. LegacyTreeData v2. University Libraries, Virginia Tech. 2023. 10.7294/22582432.

[32] Sanquetta CR, Behling A, Corte APD, Netto SP, Schikowski AB, do Amaral MK. Simultaneous estimation as alternative to independent modeling of tree biomass. Ann For Sci. 2015;72:1099–112.

[33] Singh T. Variation in ovendry wood density of ten prairie species. For Chron. 1984;60:217–21.

[34] Smyth CE, Stinson G, Neilson E, Lemprière TC, Hafer M, Rampley GJ, Kurz WA. Quantifying the biophysical climate change mitigation potential of Canada’s forest sector. Biogeosci. 2014;11:3515–29.

[35] Ståhl G, Saarela S, Schnell S, Holm S, Breidenbach J, Healey SP, Patterson PL, Magnussen S, Næsset E, McRoberts RE, Gregoire TG. Use of models in large-area forest surveys: comparing model-assisted, model-based and hybrid estimation. For Ecosys. 2016;3:1–11.

[36] Westfall JA, Coulston JW, Gray AN, Shaw JD, Radtke PJ, Walker DM, Weiskittel AR, MacFarlane DW, Affleck DLR, Zhao D, Temesgen H, Poudel KP, Frank JM, Prisley SP, Wang Y, Sánchez Meador AJ, Auty D, Domke GM. A national-scale tree volume, biomass, and carbon modeling system for the United States. Gen. Tech. Rep. WO-104. Washington, DC: U.S. Department of Agriculture, Forest Service; 2024.

[37] Woodall CW, Heath LS, Domke GM, Nichols MC. Methods and equations for estimating aboveground volume, biomass, and carbon for trees in the US forest inventory, 2010. Gen. Tech. Rep. NRS-88. Newtown Square, PA: US Department of Agriculture, Forest Service, Northern Research Station; 2011.

[38] Zeng W, Zhang L, Chen X, Cheng Z, Ma K, Li Z. Construction of compatible and additive individual-tree biomass models for *Pinus tabulaeformis* in China. Can J For Res. 2017;47(4):467–75.

[39] Zhao D, Lynch TB, Westfall J, Coulston J, Kane M, Adams DE. Compatibility, development, and estimation of taper and volume equation systems. For Sci. 2019;65(1):1–13.

